# On the Morphology of Nanostructured TiO_2_ for Energy Applications: The Shape of the Ubiquitous Nanomaterial

**DOI:** 10.3390/nano12152608

**Published:** 2022-07-29

**Authors:** Serena Gagliardi, Flaminia Rondino, Claudia Paoletti, Mauro Falconieri

**Affiliations:** ENEA, The Italian National Agency for New Technologies, Energy and Sustainable Economic Development, C.R. Casaccia, Via Anguillarese 301, 00123 Rome, Italy; serena.gagliardi@enea.it (S.G.); flaminia.rondino@enea.it (F.R.); claudia.paoletti@enea.it (C.P.)

**Keywords:** nanophase TiO_2_, dye-sensitized solar cells, perovskite solar cells, nanofluids, photoelectrochemical water splitting

## Abstract

Nanostructured titania is one of the most commonly encountered constituents of nanotechnology devices for use in energy-related applications, due to its intrinsic functional properties as a semiconductor and to other favorable characteristics such as ease of production, low toxicity and chemical stability, among others. Notwithstanding this diffusion, the quest for improved understanding of the physical and chemical mechanisms governing the material properties and thus its performance in devices is still active, as testified by the large number of dedicated papers that continue to be published. In this framework, we consider and analyze here the effects of the material morphology and structure in determining the energy transport phenomena as cross-cutting properties in some of the most important nanophase titania applications in the energy field, namely photovoltaic conversion, hydrogen generation by photoelectrochemical water splitting and thermal management by nanofluids. For these applications, charge transport, light transport (or propagation) and thermal transport are limiting factors for the attainable performances, whose dependence on the material structural properties is reviewed here on its own. This work aims to fill the gap existing among the many studies dealing with the separate applications in the hope of stimulating novel cross-fertilization approaches in this research field.

## 1. Introduction

In the ever-growing world of chemical compounds entering the nanomaterials domain, titanium dioxide is undoubtedly one of the oldest and most widely used. Starting from its former use as white pigment in paints and inks, this material has found its way into applications ranging from energy conversion to environmental and chemical engineering, sensors, consumer products and medical care [1,2,3]. As such, nanostructured TiO_2_ is presently one of the most ubiquitous and multifunctional components in the nanotechnology world. Correspondingly, a huge number of related research papers have been published in the literature, and many topical reviews have appeared to assess the current state-of-the-art and also give a historical perspective of the advancements; for instance, recent review papers have been published concerning nanophase TiO_2_ applications in photovoltaics [4] and photocatalysis [5], rechargeable batteries [6], sensors [7] and biosensors [8,9] and biomedicine [10]. Despite this ubiquity, the possibilities to tailor the material properties rely mainly on its nanophase-specific features, considering its fixed and stable chemistry and apart from the possibility of stoichiometric or compositional doping, whose functionality is still largely a matter of investigation [11]. As for its bulk properties, TiO_2_ has three crystalline polymorphs at ambient pressure, i.e., brookite, anatase and rutile [12], whose suitability for various applications is still somewhat of a matter of investigation (pp. 1–240, [13]), [14,15]; notwithstanding this situation, anatase is the most used in practice for many applications due to its ease of fabrication as nanophase and its intrinsic electronic energy properties [15]. In contrast, when it comes to the nanophase-specific features, the surface properties and the material morphology and structure add a great number of degrees of freedom and are of the utmost importance to implement specific functionalities for different applications. For example, surface termination controls the hydrophilic–hydrophobic behavior that is important for self-cleaning, photocatalytic and biological applications; moreover, it can be used to create surface trap states that are useful to control the spatial distribution of electrons and holes, which is of paramount importance in electrochemical and electronic applications. On the other hand, the morphology, i.e., the form of the basic nanophase unit forming the material, be it a bead, nanotube, needle etc., and its nanostructure, i.e., how the basic units are arranged, such as fractal aggregates, hierarchical arrangements, ordered structures etc., ultimately determines fundamental functional properties such as the optical behavior and the electronic and thermal transport. Therefore, for many applications, the material morphology and structure are the driving factors for optimized performances; this evidence constitutes the scientific basis of the present work.

According to this point of view, here we present a review on the role played by the morphology and structure of TiO_2_ in selected applications in the field of energy where the transport and optical phenomena are of the utmost importance, namely photovoltaic energy conversion by dye-sensitized and perovskite solar cells, photoelectrochemical water splitting and nanofluids for thermal management. While recent reviews [4,5,6,7,8,9,10] focused on separate and specific applications of TiO_2_, this review aims to account for the state of the art of the research describing the structural effects in such apparently different fields with a novel approach. As a matter of fact, reviews on the impact of the structural aspects of nanophase TiO_2_ on some of its applications have appeared in the literature and have mainly focused on mesoporous structures [16], thus excluding dispersions and without paying specific attention to the related transport phenomena. These aspects are instead extensively covered in the present work.

Currently, a comprehensive picture of the relationship between the material morphology/structure and its optical, electronic and thermal transport properties is yet to be achieved due to the interplay among properties and to the variety of configurations with the consequent difficulty of obtaining a realistic material description. Nevertheless, we believe that the present updated review of the experimental and, where available, theoretical works, delineating significant trends in such vast and disperse topics, can boost the exploitation of titania-nanostructures in the field of energy applications.

## 2. Photovoltaic Energy Conversion

Among the many different photovoltaic technologies, we focus here our attention only on those where nanostructured TiO_2_ is a core component, namely dye-sensitized solar cells (DSSCs) and perovskite solar cells (PSCs).

**Figure 1:**
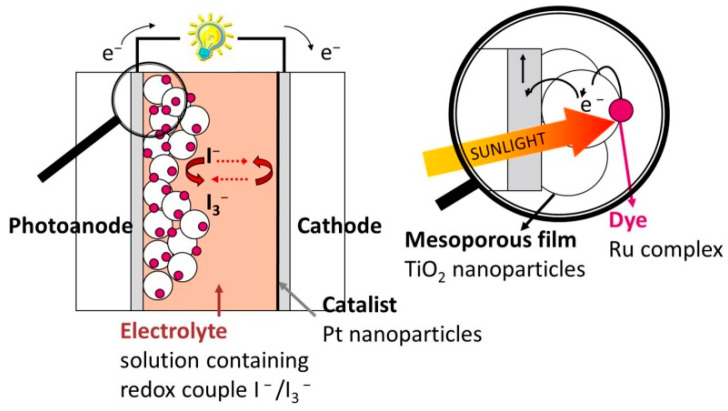
Schematics of a DSSC (**left**) and detail of the photoanode (**right**). A comprehensive review of the operating principles of a DSSC is reported in [20].

Photovoltaic energy conversion by dye-sensitized semiconductors has been widely investigated [17,18] since the last decades of the past century, but, until the publication of the paper by O’Regan and Gratzel in 1991, which reported a conversion efficiency of 7% [19], these systems typically achieved efficiency values that were too small for practical use. The disruptive idea was the replacement of a flat surface bulk semiconductor with a nanostructured photoanode (PA) with a very high surface area, allowing high dye load and consequently high light absorption. The PA in the DSSCs is a mesoporous film made of nanosized TiO_2_ beads sintered on a transparent conductive oxide (TCO) placed on a glass substrate and has a double functionality: it is both the supporting medium of the molecular light absorber (i.e., the dye) and the electron acceptor and transport layer; see Figure 1.

The first functionality requires a high specific surface area, attainable in principle by decreasing the titania nanoparticle size, and mainly determines the maximum short circuit current density *J_sc_* in the ideal condition whereby all the photogenerated carriers are collected at the electrodes, given by [20]:

(1) $$J_{SC}=q\overset{\lambda_{MAX}}{\underset{\lambda_{min}}{\int }}F\left(\lambda \right)\left[(1-R\left(\lambda \right)\right]\;A\left(\lambda \right)d\lambda$$

where *q* is the electron charge, *F*(*λ*) is the incident light spectral distribution, and *R*(*λ*), *A*(*λ*) are the device spectral reflectivity and the sensitized PA light absorption, respectively. The most used dyes, typically Ru-based organometallic molecules, allow light harvesting in the visible region and have a molar absorption coefficient of 10^7^ cm^−1^ mol^−1^ [19,21], meaning that the mesoporous titania layer thickness needed to achieve reasonable light-harvesting, for practically achievable surface areas, is about 10 μm. Even employing new organic sensitizers with higher absorption coefficients, the thinnest TiO_2_ PA is on the order of 5 μm [22,23]. Furthermore, since a large part of solar radiation is lost due to the poor dye absorption at long wavelengths, many research efforts have been devoted both to the synthesis of novel dyes with extended absorption in the infrared and co-sensitization to attain panchromatic absorption [23,24,25] to improve photocurrent and hence overall conversion efficiency. However, at the same time, techniques enhancing light diffusion phenomena within the PA have been implemented to improve the light absorption by increasing the photon path, exploiting the so-called light trapping (LT) effect. Since light scattering is strongly dependent on the dimension and shape of the scatterer, it can be argued that it can be optimized by tailoring the morphology and structure of the titania film, e.g., changing nanoparticles’ dimensions, arrangement and aspect ratio, maximizing accordingly the optical absorption in the PA and hence improving the cell performance, as widely discussed in Section 2.1.

The morphology, noticeably, also strongly influences the second functionality of PA, i.e., the electron transport toward the TCO collecting electrode [20,26]. As a matter of fact, the electron transport along the PA thickness is strongly affected by the nanostructure: the tortuous path and the electron traps related to surface states indeed limit the electrons’ mobility and constitute a bottleneck in the amelioration of the conversion efficiency of DSSCs.

Hence, huge efforts, which are detailed in the following, were devoted to the study of how the structure and the morphology of the TiO_2_ PA (i.e., dimensions and organization of nanobeads, use of hierarchical organization, use of 2D nanostructures as nanotubes and nanowires) impact on its functional properties, in order to realize high efficiency dye-sensitized solar cells.

A couple of decades after the publication of the O’Reagan and Graetzel paper [19], in 2012, the introduction by Snaith and coworkers [27] of an organometal halide perovskite extremely thin absorber infiltrated in the mesoporous titania electrode newly set up the photovoltaic research field. The perovskite absorption coefficient is one order of magnitude higher than that of organometallic dyes, so the thickness necessary for efficient light-harvesting is only some hundreds of nanometers. This down-scaling of the mesoporous titania layer thickness changed the functional requirements, as discussed in the following, meaning that the optimization strategies to achieve high conversion efficiency have been changed accordingly.

In this section, after a brief account of the theoretical background, we review selected examples of the effects of the morphology of the nanostructured titania on both the optical and transport properties of the PA in a DSSC and therefore ultimately on the device conversion efficiency; then, we discuss how some of these concepts were overcome in thinner electrodes in PSC, showing how all the knowledge achieved in more than two decades of research on nanostructured titania electrodes for DSSCs has been transferred into the development of PSCs.

### 2.1. Light Management

The increased absorption resulting from the tendency of light to be trapped by multiple internal reflections in textured surfaces semiconductors was initially exploited in silicon photodetectors and solar cells [28]. In 1981, Yablonovitch [29] proposed the first analytical approach to quantify the effect of light trapping on the interaction of radiation with scattering materials, focusing on the optical properties of media with tailored surface shapes or random surface structures. The enhancement of LT in thin film solar cells is typically achieved by a textured metal back-reflector that scatters light within the absorber layer and increases the optical path length of solar photons [30]. In DSSCs, the most commonly used dye, N719, i.e., di-tetrabutylammonium cis-bis(isothiocyanato)bis(2,2′-bipyridyl-4,4′-dicarboxylato)ruthenium(II), has a low absorption coefficient for red or longer wavelengths [21], meaning that the use of LT has become a standard practice to increase the optical path length in the cell at long wavelengths. 

**Figure 2:**
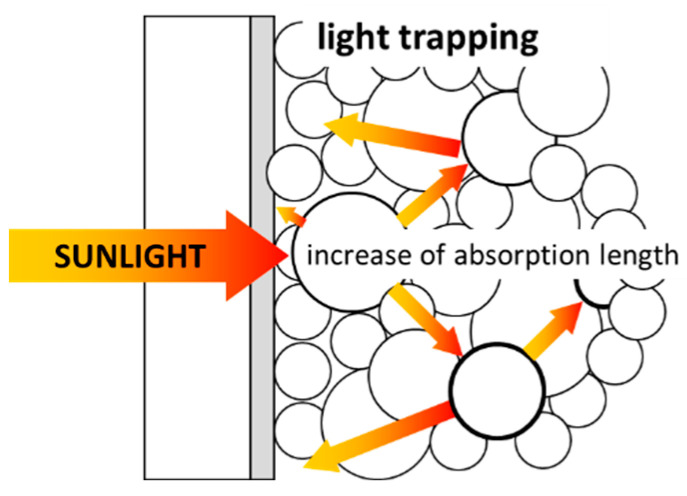
Schematic of the light trapping phenomenon in the nanostructured DSSC photoanode.

The beneficial presence of light diffusion effects in DSSCs was first theoretically analyzed by Usami [31,32] and Ferber [33]. A simple schematic of the LT process is reported in Figure 2. The exact general solution of light scattering by a particle was obtained by Mie but, since it applies to an isotropic and homogeneous sphere, it is not adequate for the TiO_2_ porous film. Usami examined the scattering of a single titania particle and then derived contributions from multiple particles using the Monte Carlo simulation method. The results indicated that optimal conditions for backscattering were consistent with the total reflection at the interface between the TiO_2_ thin film and the glass substrate. This led to the conclusion that useful optical confinement in the active layer can be achieved by inserting a reflecting TiO_2_ (rutile phase) thin film between the TCO and glass substrate and covering the PA with a film of large scattering nanoparticles. Rothemberger et al. [34] proposed a thorough optical characterization procedure for porous, thick titania electrodes and an optical model that allows the interpretation of experimental data. Moreover, they calculated a hypothetical maximum photocurrent density, assuming that each absorbed photon yields a collected electron. The optical properties of electrodes composed of a mixture of small (10–25 nm) and large (600–800 nm) particles were analyzed by Ferber. He found that the optimum optical behavior is obtained by adding 5% of larger particles with a radius of 125–150 nm to a small particles-based electrode. Gagliardi and Falconieri [35] proposed an experimental method to calculate a spectral factor to quantify the LT effect, which is useful to disentangle the concurrent light absorption by dye molecules from the scattering phenomena in the mesoporous titania film. Indeed, all the high-efficiency DSSCs based on spherical nanoparticles reported in the literature exploit LT both incorporating an additional scattering film [36,37,38] to reflect the light not yet absorbed in the active layer and embedding scattering particles in the active layer to increase the light path wavelengths [39,40,41,42] by diffusion. 

**Figure 3:**
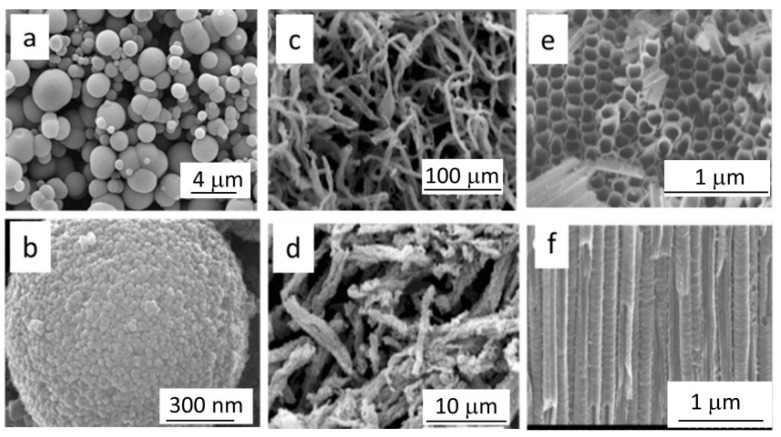
SEM images of examples of DSSCs PAs based on different TiO_2_ nanostructures. (**a**,**b**) adapted with permission from [43]. Copyright 2008, Wiley & Sons; (**c**,**d**) adapted with permission from Ref. [48], Copyright 2010, American Chemical Society; (**e**,**f**) adapted with permission from Ref. [53], Copyright 2010, American Chemical Society.

Large void spherical structures were also proposed by [43,44,45,46] to enhance light scattering within the PA, demonstrating higher light harvesting capabilities compared to small spherical nanobeads-based electrodes. Moreover, a wide variety of TiO_2_ different morphologies was used to improve LT phenomena and consequently the conversion efficiency (see Figure 3). High aspect ratio nanostructures such as nanospindles, nanowires, nanofibers and nanotubes have been proposed [47,48,49,50]; however, in most of the published literature, any explicit discussion and quantification of the impact of PA morphology on LT is missing, even though improved efficiency values are demonstrated in comparison to “conventional” PA based on small spherical particles. One of the most investigated nanostructures, i.e., titania nanotubes obtained by the electrochemical etching of titanium foils, should deserve its own discussion; here, we recall that the electrochemical process can be tuned to finely tailor the wall dimensions and length of the vertically aligned nanotubes, which grow vertically [51,52,53,54] packed in an approximately hexagonal symmetry, as visible in Figure 3. Despite the lower specific surface area with respect to those based on nanoparticles with the same thickness, nanotube PAs were believed to have superior transport properties, allowing the use of thicker films (up to tens of micrometers) to recover the dye load; furthermore, the micrometric dimensions were demonstrated to enhance the internal light scattering, thus improving light harvesting efficiency up to 20% with respect to that obtained with spherical nanoparticle-based films [51,52,53,54].

**Figure 4:**
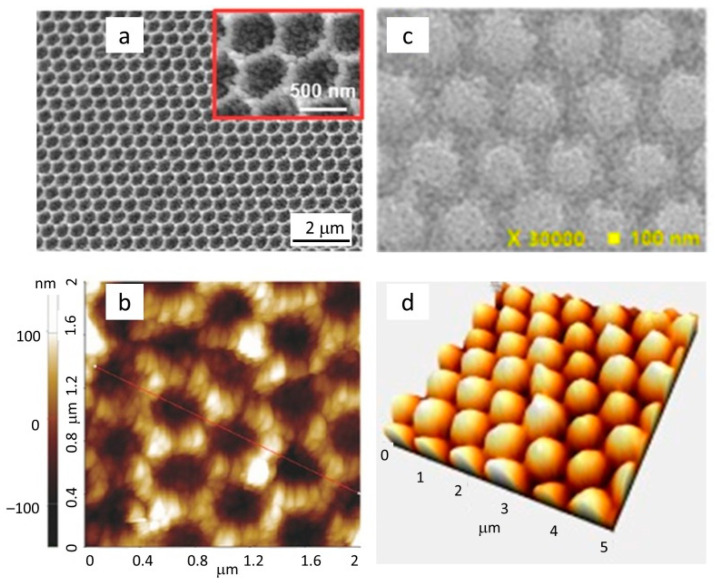
SEM (**top**) and AFM (**bottom**) images of two examples of different titania patterned electrodes in PSCs. (**a**,**b**) adapted with permission from [59], Copyright 2016, Wiley and Sons; (**c**,**d**) adapted with permission from Ref. [63], Copyright 2019, Elsevier B. V.

As mentioned before, the introduction of organometallic halide perovskites as light absorbers [27] in 2012 deeply changed the requirement of the titania mesoporous films: perovskites have an absorption coefficient one order of magnitude higher than dyes in the visible spectral region. This implies that a mesoporous titania electrode of a few hundreds of nanometers in thickness, coated with an extremely thin perovskite layer (<10 nm), absorbs 98.4% of 500 nm radiation and produces a high photogenerated current density (i.e., 17.8 mA cm^2^ in 500 nm thick layer [27]). Nevertheless, the perovskite absorption is poor in the infrared spectrum, meaning that proper photon management is still desired in order to increase the generated photocurrent. Even though the morphology of the mesoporous scaffold determines the structure and the functional properties of the perovskite-based PA absorber, the strategies developed for thick film DSSCs, such as proper choice of titania nanoparticles dimensions and/or shape and morphology engineering, are not suitable for the much thinner PA in PSCs. So, novel morphologies of the mesoporous titania scaffolds were poorly investigated for their light diffusive properties, even though 3D flower-shaped [55,56] and hollow sphere [57] nanostructures were demonstrated to improve the light harvesting efficiency by several percent in the low wavelength region. On the other hand, many studies were devoted to the texture of the interface between the TiO_2_ layer and the transparent conductive oxide in order to enhance the LT effect and accordingly improve the photocurrent: several papers report on the realization of hemispherical voids or titania structures on well-defined patterns [58,59,60,61,62,63,64]. These photonic structures maximize (see Figure 4) the long wavelength light absorption inside the cell and consequently allow higher photocurrent generation, yielding up to 13% improved overall conversion efficiencies.

### 2.2. Transport Properties

In a DSSC, electrons are photoexcited by light absorption in the dye molecules and then injected into the titania nanostructured film, where they travel until they are collected by the TCO. The nanostructured PA has a high internal surface area in contact with the electrolyte, which screens the electric field [65], meaning that the travel of the electron from a nanoparticle to another is a random walk between equivalent sites and may be considered a diffusive process [66,67,68]. This picture is consistent with the experimental values measured for the electron diffusion coefficient *D*, which is found to be one or two orders of magnitude lower than that expected for a bulk single crystal of TiO_2_ [69,70]. For this reason, understanding the dependence of the electron transport efficiency in the porous film on the network topology [71,72] and on the grain morphology [69] is of uppermost importance for the improvement of solar cells conversion efficiency. To this end, several papers discuss the electron transport using models based on a random walk approach, in which the electron diffusion coefficient *D* is related to the film porosity *P* according to the percolation theory [71]:

(2) $$D=a{\left|P-P_{C}\right|}^{\mu }$$

where *a* is a constant containing information about the average distance between connected particles and the electron residence time in a particle, *P_C_* is the critical porosity above which the film is not continuous, and *μ* is the power-law exponent associated with the conduction mechanism. As the film porosity increases, the coordination number decreases, leading to a tortuous and longer electron pathway, increasing the number of particles visited by electrons and increasing the fraction of dead-end particles. Transient photocurrent measurements confirm the model predictions, obtaining values of *D* that decrease from ≈5·10^−3^ cm^2^ s^−1^ to ≈5·10^−4^ cm^2^ s^−1^ at 1 sun illumination when the porosity increases from 0.5, i.e., the typical value in DSSC PAs, to 0.8, close to the critical porosity value. On the other hand, the value obtained for the power-law coefficient by fitting the experimental data with the percolation model is much lower than that expected for random mixtures, suggesting that a rearrangement of the nanoparticles occurs during the sintering process, preventing the formation of isolated clusters. 

On the other hand, the experimental observation of slow transport, with time constants ranging from milliseconds to seconds and a small diffusion coefficient, both depending on the illumination intensity [73,74], shows the existence of a trap-limited transport mechanism [66,75,76,77,78,79,80,81,82,83,84,85]. This transport mechanism, due to electron trapping and detrapping processes in an exponential density of band gap states, was actually demonstrated with different experimental techniques, such as transient photocurrent measurements [66,73,78,86], intensity modulated photocurrent spectroscopy [73,74,77,78,81,87] and current–voltage measurements [67,77,82].

With the aim of improving the overall conversion efficiency of DSSC by optimizing the conductive properties of the PA, the spherical nanoparticles were frequently substituted with randomly organized elongated nanostructures, such as nanowires [88,89], hollow fibers [48] and nanospindles [49] or also well-ordered vertically oriented nanotubes [90,91]. For electrochemically synthesized and vertically oriented nanotubes, the morphology implies a significant reduction in the specific surface area and hence the necessity of much thicker PAs to achieve comparable dye load. Amorphous titania nanotubes are grown by titanium anodization, and the crystallization is achieved by subsequent thermal annealing in oxygen. Growing titania nanostructures from a Ti foil gives a non-transparent anode which is to be implemented in the so-called back-illuminated DSSCs, where the light losses due to undesired light absorption in the counter electrode and in the electrolyte decrease the generated photocurrent; to avoid this geometry, Ti films were first deposited on the transparent conductive oxide, then anodized and finally annealed. In this way, nanotube-based PAs were realized and implemented in DSSCs. Nevertheless, the efficiency of these solar cells was not much better than those realized with conventional electrodes, even though PA thickness was reported to reach 33 μm, thus compensating for the otherwise small dye load [90,91]. The unexpectedly poor performance as the DSSC anode of the ordered nanotubes arrays was attributed to the deterioration of the TCO occurring during the thermal treatment of the titania nanotubes but also to the high density and deepness of the traps that degrade the transport properties of the film, pointing out the need for a technical improvement in the realization steps.

Titania nanotubes were also mixed with nanoparticles after detachment from the titanium foil, and the PA made of this mixture was demonstrated to perform slightly better than a conventional PA only for a relatively small number of nanotubes (10 wt%) [92]; indeed, for high concentrations of titania nanotubes, the structure becomes highly disordered, resulting in longer electron pathways, which degrade the overall transport properties of the electrode. Ghadiri et al. [48] proposed a hollow nanofiber PA morphology, which has transport properties superior to those based on nanoparticles, because the nanofibers have a lower number of contact points and hence a less tortuous electron path and a lower number of traps. Since the quality of the contact points between nanostructures affects the overall transport properties, nanospindles, having low-defect single-crystalline faceted surfaces, were synthesized, demonstrating better transport properties when used in DSSC PAs and improving the overall conversion efficiency [49].

Indeed, the trap-limited transport mechanism usually overwhelms the role of the structural order in the transport properties; thus, Villanueva et al. [93] proposed an approach to evidence the structural effects free from the interference of transport-limiting traps. To this end, they conveniently described the diffusion coefficient *D* as a function of the freely mobile electron diffusion coefficient *D*_0_ by the relation [93]

(3) $$D\sim D_{0}{\left(\frac{N}{N_{C}}\right)}^{\left(\frac{1}{\alpha }-1\right)}$$

where *α* is a parameter describing the “energetic disorder” (related to the number of localized states in the band gap: i.e., the smaller is *α* the higher is the disorder), *N* is the total photoelectron density, and *N_C_* is the effective density of trap states at the transport (mobility) edge. While *D*_0_ is unaffected by the energetic disorder associated with the exponential distribution of band gap states and surface traps, it depends on the structural disorder, i.e., grain size, number and surface area of the particle boundaries. With the proposed approach, it is possible to determine the dependence of *D*_0_ on the square of the particles’ size and demonstrate that the transport velocity can decrease by two orders of magnitude from a bulk situation, with *D*_0_ ranging between 0.1 and 0.5 cm^2^ s^−1^ (when the particle size tends to the micrometer film thickness value) to small nanoparticles, with *D*_0_ ≈ 10^−3^ cm^2^ s^−1^ (diameter down to few nanometers), while trapping effects can have an impact up to five orders of magnitude on the transport time. This observation shows the necessity of the development of high-quality crystalline structures, where direct electron pathways, enabling good transport properties (with the high specific surface area necessary for high dye load), allow highly efficient energy conversion, which is even more relevant in low illumination conditions, such as in indoor applications.

**Figure 5:**
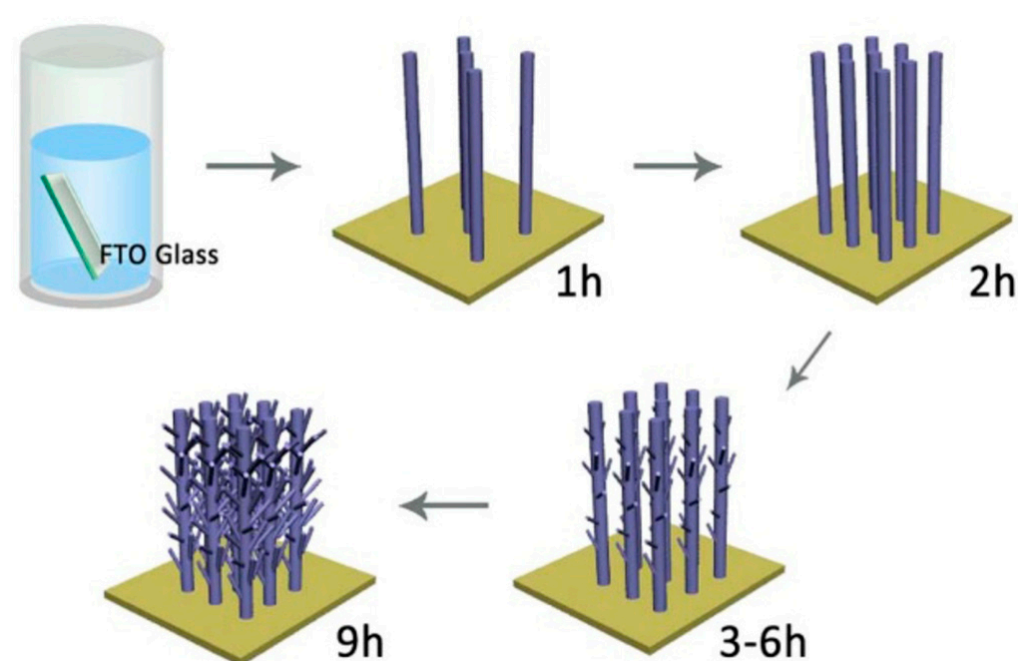
Schematic of formation process of hierarchical TiO_2_ nanostructures. Reproduced from 354 Reference [97] under the Creative Commons CC-BY-NC-ND license.

For this purpose, hierarchical nanostructures, constituted by long nanotubes or nanowires, decorated with nanoparticles or nanobranches, were therefore synthesized and used as mesoporous PA in DSSCs [44,94,95,96,97]. Results reported by Qu et al. [94] demonstrated that, with respect to PAs based on nanoparticles, those made of hierarchical structures showed improved charge transport properties, due to the low trap density in the highly crystalline structure and short electron path (see Figure 5), but also the specific surface area was still low and needed to be optimized for the generation of high current density. On the other hand, Wu et al. [46] demonstrated that processes leading to the formation of small nanobranches on nanorod structures, even if producing high surface area PA with a high short circuit photocurrent, deteriorate the charge transport properties, inducing the formation of a large number of defects. Thus, even if the use of hierarchical structures paved the way for high efficiency DSSCs, the optimization of the functional properties of the mesoporous films is still necessary for their practical exploitation.

As discussed above, the high absorption coefficient of the perovskites reduces the necessary PA thickness in PSCs down to a range between 400 nm and 1.5 μm [96]; this small thickness value immediately relaxes the requirements for the charge transport properties in the titania layer. Nevertheless, the morphology of the TiO_2_ film strongly influences the overall performance of PSCs. It was indeed demonstrated that the porosity of the titania scaffold, which mainly depends on the dimensions of spherical nanoparticles, determines both the perovskite coverage and crystallinity [98,99,100,101,102,103]. Improved coverage of the titania scaffold, obtained by increasing the porosity, gives rise to better light harvesting capabilities and hence results in higher short circuit current density generation [101,102,104]. Another consequence of the improvement of the TiO_2_ scaffold coverage is the reduction of undesired electron–hole recombination, which occurs at the interface between electron and hole-transport layers; accordingly, a more effective capability of charge extraction from the perovskite was demonstrated by measuring the photoluminescence intensity, which decreases for increased titania film porosity while maintaining the same thickness. It was also reported that when maintaining the same morphology, when the titania film thickness exceeds an optimal value, the transport characteristics of the electrode [104] degrade because the perovskite coverage decreases, allowing recombination processes, and also, as widely discussed above, the number of interparticles connections decreases, lengthening the electron path. Moreover, many papers report on the impact of the perovskite grain size on the charge recombination processes not explicitly addressing the role of the titania scaffold morphology [100,105,106] but showing the improved performance of cells with larger grains, which are less affected by trap-assisted recombination at the grain boundaries. 

Aside from the interest in the optimization of titania scaffolds based on spherical nanoparticles, many papers report on the performance achieved with completely different nanostructures. On one hand, the nanotubes and nanorods ordered structures that, while being unsuitable as dye-sensitized PA with submicrometer thickness for the low surface area (and hence low dye load), were demonstrated to allow good performance when used as a perovskite scaffold [107,108,109,110,111]. On the other hand, a wide variety of mesoporous films created by disordered nanowires and nanofibers [112,113] or even hierarchical heterostructures [114,115,116] showed good performance in PSCs. 

The results presented in this review represent the evolution and the latest progress related to the use of titania mesoporous films in dye-sensitized and perovskite solar cells. However, research is still in progress in both the fields because of the high expected impact that their commercialization would have on the sustainable production of energy. Indeed, thanks to their high conversion efficiency under dim light (up to 32%) [23,24,25], DSSCs are promising devices to recycle the consumed electric power in indoor applications; on the other hand, the high conversion efficiency demonstrated by PSCs paves the way for their commercialization for high-power photovoltaic plants. Moreover, the capability of both DSSCs and PSCs to be implemented in thin flexible modules [117,118] with a pleasant aesthetic appearance makes them the ideal candidates for both portable electronics and building-integrated photovoltaics [4,119].

## 3. Photoelectrochemical Water Splitting

One of the most studied applications of nanophase TiO_2_ in green energy production is its use as a photoanode for hydrogen generation through the light-driven decomposition of water in a photoelectrochemical (PEC) device [120,121,122,123].

Titanium dioxide presents many of the characteristics of an ideal semiconductor, such as chemical and biological inertia, photocatalytic stability and ease of production. Moreover, it is cheap and free of risks to human health and the environment; noticeably, for PEC applications, the most important drawback of TiO_2_ is that it can be activated only by ultraviolet light, so only about 4% of the solar spectrum can be used in solar-driven generators. In fact, photoexcitation of TiO_2_ occurs when the impinging photon energy is greater than the semiconductor bandgap, which is equal to 3.2 eV and 3.0 eV for the two most common crystalline phases of pure TiO_2_, i.e., anatase and rutile, respectively. Given the focus of this review on morphology, we do not discuss here the most studied strategies adopted to develop visible light responsive photocatalysts, such as doping or modification of the surface with plasmonic nanostructures or dyes [124,125,126,127]; instead, we show how the optical efficiency can be improved by exploiting light-trapping effects, referring to the theoretical background given in the previous section. 

When immersed in water, TiO_2_ is very stable, and its photoexcitation can drive a redox process in the interfacial region between the fluid and the semiconductor, since the electron-hole (e^−^/h^+^) pair that is created is energetically rich and can activate chemical reactions according to the following paths:

TiO_2_ + 2*hν* → 2e^−^ + 2h^+^ (4.1)

2h^+^ + H_2_O → ½O_2_ + 2H^+^ (4.2)

2e^−^ + 2H^+^ → H_2_ (4.3)

**Figure 6:**
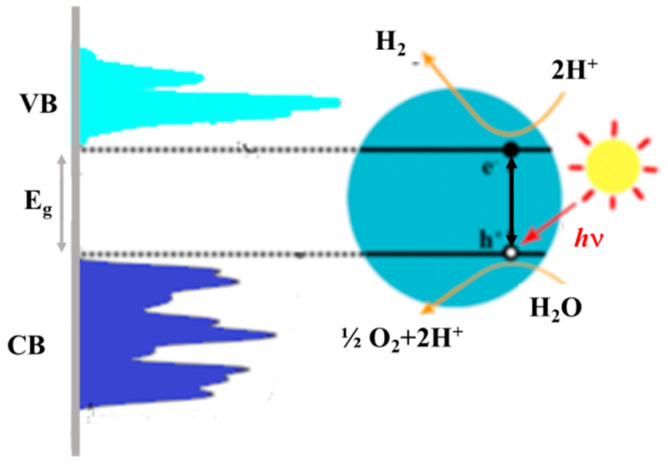
Scheme of the key steps of the photocatalytic reaction in a titania nanoparticle. If the incoming photon has an energy *hν* greater than the value of *Eg* (energy gap or band gap), an electron (e^−^) can be promoted from the valence band (VB) to the conduction band (CB), leaving behind a hole (h^+^). The electronic structures of VB and CB were determined from X-ray Absorption and Emission Spectroscopy. Adapted with permission from [132]. Copyright 2014, American Chemical Society.

Separation of the photogenerated e^−^/h^+^ pairs occurs in the semiconductor because of the electric space-charge field associated with the band-bending, which depends on the presence of surface states and the difference between the redox potential of the electrolyte and the Fermi level of the semiconductor [128,129]. The energetic holes, transported at the semiconductor surface, can effectively participate in the water oxidation reaction with consequent dissociation and hydrogen/oxygen evolution [1,130,131]; see Figure 6.

**Figure 7:**
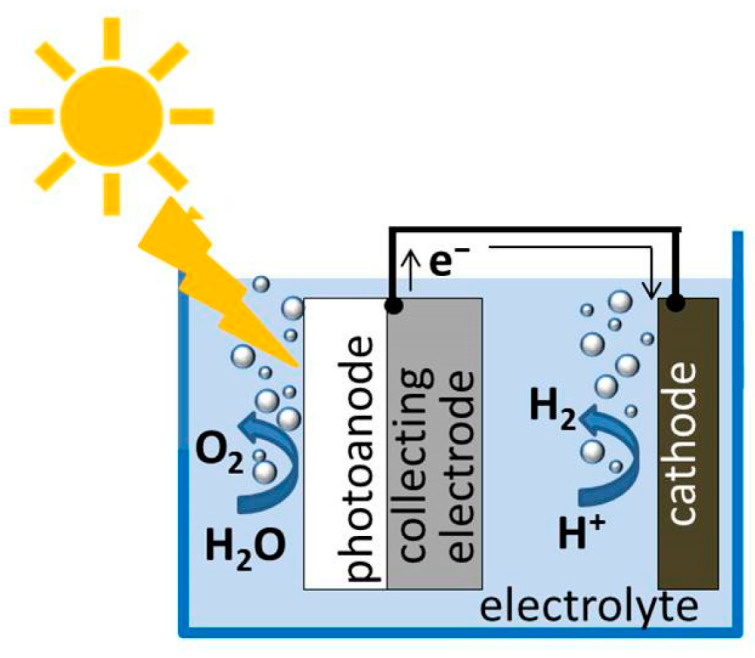
Schematics of a photoelectrochemical cell for water-splitting. The light impinging on the photoanode generates hole and electron pairs; the first migrate to the surface and promote the oxygen evolution, while the second, after travelling to the cathode, drive the hydrogen evolution.

Photoinduced hydrogen production from water splitting can also be obtained in a PEC cell, where the main components are the semiconductor light-absorbing photoanode, the water-based electrolyte and the cathode (see Figure 7) [133], this process being more interesting for long-term applications with respect to the photocatalytic operation, as it provides gas separation and post-operation material recovery.

In the PEC cell described above, three major physicochemical steps are involved [134]. The first step is the light absorption by the semiconducting (n-type) photoanode, i.e., TiO_2_ in our case of interest, which produces pairs of charge carriers; the second step is the separation and transportation [135] of the photogenerated pairs: electrons travel through the photoanode to the collecting electrode contact, while holes travel towards the electrode/electrolyte interface. The last step consists of the surface redox reactions at the electrodes, namely water oxidation (reaction 4.2) at the photoanode and proton reduction (reaction 4.3) at the cathode, resulting in water splitting. An efficiency parameter can be related to each of these three steps so that the overall efficiency, *η_overall_*, of PEC water splitting can be expressed as [136]

(5) $$\eta_{overall}=\eta_{e^{-}/h^{+}}\cdot \eta_{transport}\cdot \eta_{interface}$$

where $\eta_{e^{-}/h^{+}}$ is the efficiency of e^−^/h^+^ photogeneration, *η_transport_* is the efficiency of charge transport, and *η_interface_* is the efficiency of surface reactions. Experimentally, to evaluate the performance of a PEC PA, the most common practice is to measure the photocurrent under illumination, because it is proportional to the hydrogen or oxygen production rate [123]. Indeed, the solar-to-hydrogen conversion efficiency is proportional to the photocurrent circulating in the external circuit according to the relation [123]

(6) $$\eta_{overall}=\frac{V_{redox}J}{P}$$

where *J* is the photocurrent density (in A m^−2^), *P* is the power density of illumination (in W m^−2^), and *V_redox_* is the potential corresponding to the Gibbs free energy change per photon required to split water (which is equal to 1.23 V).

Since the first PEC cell with a TiO_2_ PA was assembled [130], the search for materials and experimental conditions that better fulfill the thermodynamic and kinetic requirements for efficient water splitting photoconversion has been underway. In this respect, nanostructured electrodes present undoubted advantages. The first benefit is due to the capability of nanostructures to improve the coupling with incoming photons by LT, eventually producing an optical absorption enhancement (see Section 2.1); the second is the possibility of decoupling the direction of light absorption and charge-carrier collection; that is, instead of diffusion through the bulk material, orthogonal separation of photogenerated charges occurs, and the probability of charge recombination is reduced [137]; finally, the third advantage achieved by nanostructuring is the huge increase of the surface area [138,139] that, enlarging the number of reaction sites [140], improves *η_interface_*. In the following, in line with the scope of this review, we discuss how titania morphology and structure impact the functionality of the electrodes in PEC devices and report the main published experimental results.

### 3.1. Light Management

**Figure 8:**
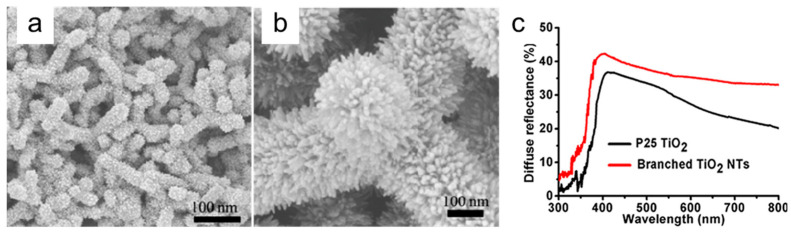
SEM images of hierarchical titania nanostructures (**a**,**b**), and diffuse reflectance spectra (**c**) of electrodes made of commercial titania nanoparticles (black curve) and of hierarchical nanostructures (red curve). Adapted with permission from [142]. Copyright 2014, American Chemical Society.

Following the photon absorption process, the carrier photogeneration efficiency $\eta_{e^{-}/h^{+}}$ is expected to increase with the TiO_2_ film thickness, until this is within the effective region of photon penetration, which spans from ≈0.3 to 10 μm in the UV region from 330 nm to 370 nm depending on the photon wavelength [141]. In the visible part of the solar spectrum, titania is only slightly absorbing, so, as in the photovoltaic applications (see Section 2.1), LT occurring in the nanostructured electrode can be exploited to enhance absorption with the ultimate effect of improving the conversion efficiency under solar irradiation. In water splitting applications, only few papers report on light management strategies adopted to improve the performance of PEC cells. In particular, Ibadurrohman et al. [135] studied the behavior of PEC cells based on mesoporous electrodes made of nanoparticles and attributed part of the conversion efficiency improvement to the surface roughness induced by the use of a PEG template in the synthesis of the titania film, even though they could not disentangle LT effects from charge separation and transport ones. More explicitly, Qiu et al. [142] discussed, in terms of LT within the mesoporous structure, the higher conversion efficiency in electrodes made of hierarchically branched anatase TiO_2_ nanotetrapods with respect to nanoparticles-based ones, showing that the former structure has better light diffusing properties (see Figure 8). The improved efficiency due to the occurrence of LT when using 1D structures is also reported in Yu et al. [143], Shankar et al. [144] and Ozkan et al., who pointed out the role of the interspacing of TiO_2_ nanotubes (tube-to-tube spacing) in the light diffusion process [145]. 

In addition, as outlined for PSCs (see Section 2.1), a nanostructured interface between the collecting electrode and the titania film was demonstrated to efficiently trap solar radiation; in [142], aluminum nanospikes were created on the TCO surface, while Chiarello et al. [146] showed that ordered titania nanotubes in a photonic crystal structure confine light of a selected wavelength in the region of the walls, exploiting the periodical modulation of the refractive index to enhance the light absorption.

### 3.2. Surface Reactivity and Electron Transport

As already mentioned, the efficiency of surface reactions (*η_interface_*) is strictly linked to the number of reaction sites and the PA surface area. The simplest and in principle most efficient strategy to enlarge the specific surface area of the PA is depositing small nanobeads (0D structures) on the collecting electrode [147,148]. This kind of film exhibits a huge increase in the specific surface area for decreasing particle diameter. In addition, the small nanoparticle dimension allows the photogenerated holes to reach the surface with a very short travel distance, thus minimizing undesired recombination processes. Nevertheless, a minimum nanoparticle size (≈1 nm) is required because the photocatalytic activity decreases with decreasing size, as demonstrated by a theoretical study showing that the water splitting reaction in very small beads is thermodynamically disadvantaged because of the strong self-trapping of free electrons and holes [149]. Experimentally, it was demonstrated that maximum photocatalytic efficiency is obtained for particle dimension of about 25 nm [150], which is a trade-off between optical requirements (larger particles absorb light more efficiently) and the necessity of maximizing the ratio of the e^−^/h^+^ pairs at the interface to total generated ones (which increases as the particle size decreases). 

**Figure 9:**
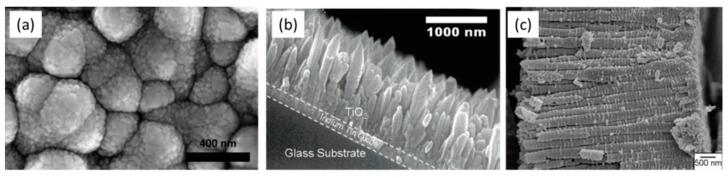
SEM images of different titania nanostructures for PEC electrodes: rough nanobeads (**a**) adapted with permission from [135], Copyright 2015, American Chemical Society, columnar (**b**) adapted with permission from [160], Copyright 2008, American Chemical Society, and tubular structures (**c**) adapted with permission from [144], Copyright 2009, American Chemical Society.

As thick PAs are desirable to attain high photocurrent density and improve overall cell efficiency (because the number of reaction sites and the optical absorption grow), many efforts have been devoted to developing highly efficient PEC cells based on thick Pas. One of the main bottlenecks presented by thick films is constituted by the difficult access of the electrolyte to the pores through the whole electrode, so different strategies were adopted in the preparation of the film to control and optimize the porosity. In sol-gel methods, the addition of surfactant was used to control the pore (and titania crystallite) dimensions, achieving properly porous thick films (thickness up to ≈1.2 μm) with improved photocatalytic activity [151]. In spray pyrolysis preparation methods, highly porous and rough films were realized by using a templating agent (see Figure 9), attaining an improved conversion efficiency of a factor 5 [135] with respect to PAs made of unmodified titania beads. On the other hand, for electrosprayed films, the structure [152] is mainly determined by the nanoparticle aggregation in the starting suspension. For such kinds of electrodes, it was demonstrated that large pores are created by depositing large clusters. Nevertheless, a detrimental reduction of hydrogen generation for excessively porous films was observed [153,154]; this phenomenon can be attributed both to the long and tortuous path of the electrons, leading to undesired recombination processes, and also, as reported for DSSCs electrodes [155], to a tortuous path for ion diffusion. For this reason, a trade-off between high porosity, leading to high surface area and easy electrolyte access, and short electron (and ion) paths are necessary to reach a high hydrogen production rate.

Unlike what happens in a photocatalytic reactor, in a PEC cell, the fate of photogenerated holes and electrons is quite different: while the first, as in photocatalysis, only need to reach the nanoparticle surface to react with the electrolyte, electrons must travel to the collecting electrode through the nanostructured titania PA. Therefore, the hole diffusion length of about 10–70 nm measured in nanostructured TiO_2_ [15,135,156,157,158] is quite sufficient to guarantee the hole transport efficiency close to unity, while the electron transport could represent a bottleneck for the overall hydrogen production efficiency [159]. Electron transport in mesoporous electrodes made of almost spherical nanoparticles has been widely studied in DSSCs, as reported in Section 2.2, and some papers demonstrated that, also in water-splitting PEC cells, the electron travel is random walk-like and subject to multiple trapping and detrapping events [80,141].

A useful parameter to quantify the competition between the diffusive transport and the recombination process is the electron diffusion length, which is the average displacement of an electron before recombination. In a PEC cell, the electron diffusion length of a nanoparticle-based PA was measured under illumination by UV light (wavelength between 330 and 390 nm), obtaining values ranging from. 8.5 to 12.5 μm [141]. These experimental results showed that for electrode thicknesses up to a few micrometers, i.e., optimized for UV light harvesting, the electron collection is quite efficient. Nevertheless, thicker PAs are necessary to increase both the reactive area and the exploitation of the solar spectrum by extending light harvesting to visible, which is poorly absorbed in titania [141]; hence, when increasing the electrode thickness up to 10 micrometers or more, the requirements on the electron diffusion length become more severe.

To fulfil this necessity, so far, one-dimensional (1D) nanostructures (see Figure 9) such as columnar single crystals [160] nanotubes [161,162,163], nanowires [164,165,166], nanofibers [167,168,169,170,171] and nanorods [170,171] have been extensively studied for the superior electronic properties (longer charge carrier lifetimes and shorter transport times), as widely discussed in Section 2.2. 

Lately, the most investigated morphology of nanostructured TiO_2_ PAs is the tube-shaped one, vertically aligned with respect to the collecting electrode. In such kinds of electrodes, photogenerated holes only need to move radially across the nanotube wall to react at the solid/electrolyte solution interface, while the electrons follow the direct conduction pathways along the wall, improving the charge collection [172]. A great effort has been spent on the optimization of the morphology of nanotubes, since PEC tests showed that besides crystallinity, length, pore diameter and wall thickness critically affect photoelectrochemical activity [173,174]. Indeed, PAs made of nanotubes with small pore diameters exhibit low photocurrent, and the phenomenon is explained by the hindered diffusion of the electrolyte solution into the tubes [151,175,176,177] and the higher charge transfer resistance. As regards the wall thickness, thin-walled (3–5 nm) nanotubes facilitate the diffusion of the photogenerated holes to the semiconductor/electrolyte interface during water splitting, allowing for efficient separation of charges [145,176]; also, porous-wall nanotubes were used in PEC devices, showing a good photoconversion efficiency [178], thanks to the increased surface area. 

Regarding the tube length, many papers report on the study of the optimal length to obtain a high PEC performance, but a wide consensus on this parameter is still missing due to discordant results, even though it is clear that a compromise between *η_interface_* and *η_transport_* is needed [175]. Generally speaking, the 1D nanostructuring is less effective than 0D for the increase of the specific surface area; so, if compared to electrodes made of 20 nm diameter beads, electrodes made of nanotubes (wall thickness 20 nm, inner diameter 10 nm) should be thicker by a factor of 2 to recover the same specific surface, but increasing the tube length affects electrolyte diffusion and enhances the requirements on conduction properties, although improving the light absorption at longer wavelengths. So, several works [179,180,181] have reported that the increase in TiO_2_ tube length provides higher PEC activity by enlarging the active surface area, thus increasing the number of the reaction sites and also offering higher absorption of the incident light. In this direction, a remarkable photoconversion efficiency of 16.25% was achieved under UV illumination in a PEC with an electrode made of nanotube arrays long up to 30 μm with suitable wall crystallinity [182]. In other works, the photocurrent density was proven to decrease with increasing tube length, and the effect was explained in terms of the higher chances of the e^−^/h^+^ pair recombination for the longer paths. For instance, a significant decrease in photocurrent density was found when the nanotube length was higher than about 5 μm [179], while the highest performance (50 μA cm^−2^) was recorded for nanotubes not longer than 2 μm [183] and 1 μm [161]. These discordant results can be ascribed to the different transport properties of the tubes, due for example to crystallinity, trap density and quality of the interface with the collecting electrode; all these characteristics are strictly related to the preparation process, which ultimately determines the optimal tube length. Thus, characterization protocols enabling the disentanglement of the length on the functional behavior of the PA from the synthesis procedure and the direct comparison of the published results could greatly boost the optimization of novel electrodes. 

**Figure 10:**
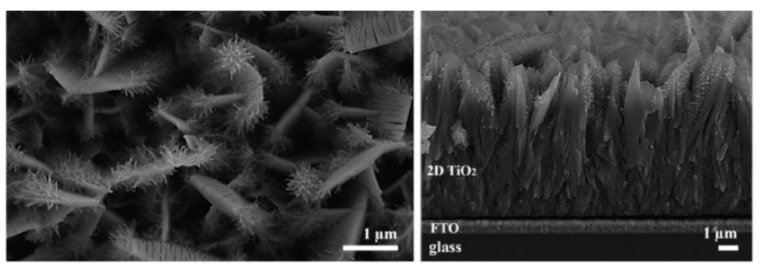
SEM images of branched tubular titania structures. Adapted with permission from [184], Copyright 2018, Wiley.

To exploit the direct electron paths in 1D structures, while increasing the surface area, tubular TiO_2_ PAs with additional branches grown out from the plate-like backbone structure have been studied (Figure 10). They present excellent charge separation and transport efficiency and also provide a highly accessible surface area, as the branches act as direct pathways to efficiently extract holes from the bulk structure to the semiconductor/electrolyte interface, which results in faster separation of photogenerated e^−^/h^+^ pairs and facilitates reactions at the semiconductor/electrolyte interface [184,185]. Moreover, the photogenerated electrons are transported efficiently via the single-crystalline backbone nanostructure as the direct pathways to counter electrodes for proton reduction. The use of this kind of structure leads to a high photocurrent of up to 1.02 mA cm^−2^, which is close to the theoretical upper limit of photocurrent density of anatase TiO_2_ (1.1 mA cm^−2^) [184,185]. Furthermore, it was argued that the direct growth of this nanostructure on TCO provides an intimate contact between the film and substrate and improves the electron collection efficiency [185].

Other studies present an attractive alternative, building a hierarchically porous system joining macropores within the mesoporous films, which can lead to more accessible pore openings and increase the availability of the internal surface. PAs constituted of hierarchically macro/mesoporous TiO_2_ films prepared by a template synthesis reveal excellent performance in PEC water-splitting due to a high surface area and hierarchical pores with 3D interconnected highly crystalline anatase frameworks [186].

Yang et al. [187] report 3D nanostructured hierarchically TiO_2_ photoanodes characterized by a core-shell structure: these structures combine nanodendrites in a core portion and a shell portion formed by nanoparticles sequentially located on the surface. The inner TiO_2_ array provides a fast electron transport pathway due to its quasi-single-crystalline structure, while the nanoparticles in the shell portion provide a larger surface area for more efficient charge separation without significantly sacrificing the electron collection efficiency. Furthermore, the combination of crystalline rutile and anatase phase in a 3D hierarchical structured PA shows a remarkable photocurrent density of 2.08 mA cm^−2^ thanks to the combination of crystalline phases of rutile/anatase in the 3D structure (the maximum photocurrent for rutile TiO_2_ at 1.23 V vs. reversible hydrogen electrode under simulated sun light at 100 mW/cm^2^ was calculated to be 2 mA/cm^2^) [187].

Considering the current state, PEC technologies are still far from meeting the demand for successful industrialization. Nevertheless, the increasing global demand for sustainable and environmentally friendly energy production encourages further research on solar-driven hydrogen production. In particular, there is a large consensus on the benefits of both the development of novel nanophase photocatalysts and of new architectures of the heterojunction between TiO_2_ and cocatalysts. Further studies are expected to overcome the main bottlenecks, such as low utilization of solar energy and high cost of synthesizing materials, paving the way for the scaling-up of the low-cost production of high performance devices. 

## 4. Thermal Energy Management by Nanofluids

Nanofluids (NFs) are colloidal suspensions of nanoparticles (beads, nanofibers, nanotubes, etc.) dispersed in a base fluid, generally water, ethylene glycol or oil, where the nanophase has the function of modifying the thermal transport properties of the base fluid. Among all the nanoparticles with suitable thermal properties, including metallic and nonmetallic ones, TiO_2_ nanoparticles are most commonly used, due to the stability of their chemical structure, their biocompatibility and their electrical, optical and physical properties [188,189,190,191].

NFs have attracted particular interest because of their capacity to increase heat transfer efficiency in coolant applications, showing higher thermal conductivity values than those predicted by the classical models for composites [192,193,194,195,196]. These models, derived from the Maxwell theory [197] for electrical conductivity of composites, are essentially based on the Effective Medium Approximation (EMA) [198,199,200] and describe stationary systems characterized by the conductivity of phase constituents (base fluid, *k_l_,* and nanoparticles, *k_p_*) and the nanoparticle volume fraction (*φ_p_*), originally dealing with spherical inclusions. Later, in the Hamilton and Crosser (HC) model, ellipsoidal particles were included through the introduction of a sphericity parameter *n* [201] with values ranging from 3 to 6 for spherical to ellipsoidal particles; in this case, the effective thermal conductivity is expressed as

(7) $$\frac{k}{k_{l}}=\frac{k_{p}+\left(n-1\right)k_{l}+\left(n-1\right)\varphi_{p}\left(k_{p}-k_{l}\right)}{k_{p}+\left(n-1\right)k_{l}-\varphi_{p}\left(k_{p}-k_{l}\right)}$$

Further refinements of the basic EMA model considered the contribution of the interfacial thermal resistance (*R_b_*) between the composing phases for spherical and ellipsoidal particles [202,203]. Indeed, although *R_b_* can be considered negligible for microsized particles, for nanosized inclusions, its impact should be pronounced due to the larger surface area, which leads to a reduction in the thermal conductivity of the composite system [204]. On the other hand, Wilson et al. [205] and Huxtable et al. [206] experimentally measured the *R_b_* between different nanoparticle/fluid couples, obtaining values ranging from 0.77·10^−8^ Km^2^ W^−1^ to 20·10^−8^ Km^2^ W^−1^. Compared with solid/solid interfaces, such low thermal resistance values cannot limit the heat transfer and affect the thermal conductivity in NFs [207]. 

Diversely, other theoretical approaches considered the particle/fluid system as a dynamic system, where the heat transport is favored by the convection mechanism induced by the Brownian movement (BM) of the nanoparticles [208,209,210,211]. For instance, Kumar et al. [212] extended the stationary model to include the effect of the nanoparticle motion in terms of particle velocity as responsible for the thermal conduction, demonstrating its key role in determining the temperature dependence of the heat conduction. Later, Prasher et al. [213] proposed a Brownian motion-based convective–conductive model to explain the behavior of the NFs with respect to temperature and nanoparticle concentration and size, even though some opposing results evidenced that the BM does not have much influence on thermal conduction [214,215,216]. 

Notwithstanding the numerous theoretical approaches present in the literature to predict the thermal conductivity enhancements measured in NFs, none can reproduce the observed thermal conductivity enhancements in the full achievable range of particle size and temperature, so this is a currently very active area of research. 

Experimentally, for TiO_2_-based NFs, different thermal conductivity enhancements with respect to the base fluid are reported in the literature: for instance, typical enhancements of 2–3% were observed at low nanoparticle volume fractions (<1%) [217,218,219,220] in similar temperature range and nanoparticle size conditions (10–30 nm), while other researchers measured larger thermal conductivity enhancements up to 20% [193,221]. Reasons for these discordant values can be traced both to the different experimental techniques used for the thermal characterization and, more interestingly, to the different NFs’ physical and chemical characteristics [222,223]. As for the experimental techniques, for example, the most common methods used to measure the effective thermal conductivity of nanoparticle suspensions, such as the transient hot-wire method [224], the steady-state parallel method [217,225], the temperature oscillation method [226] and the hot strip method [227], can influence the natural thermal convection of the base fluid by adding additional heating that can affect the measurement results. Diversely, the forced Rayleigh light scattering method [220,228,229] is a contact-free method with a very short measuring time that limits the temperature rise during the measurement, leaving the system conditions unaltered. On the other hand, as mentioned before, the reported thermal conductivity differences are also a result of different NF characteristics such as nanoparticle dimensions and morphology, polydispersity, volume fraction and the presence and structure of aggregates, but also the base fluid pH value, which, for instance, influences the particle clustering [230,231,232] and/or the liquid layering at the nanoparticle/liquid interphase [202,203,233,234,235], significantly modifying the thermal conduction.

**Figure 11:**
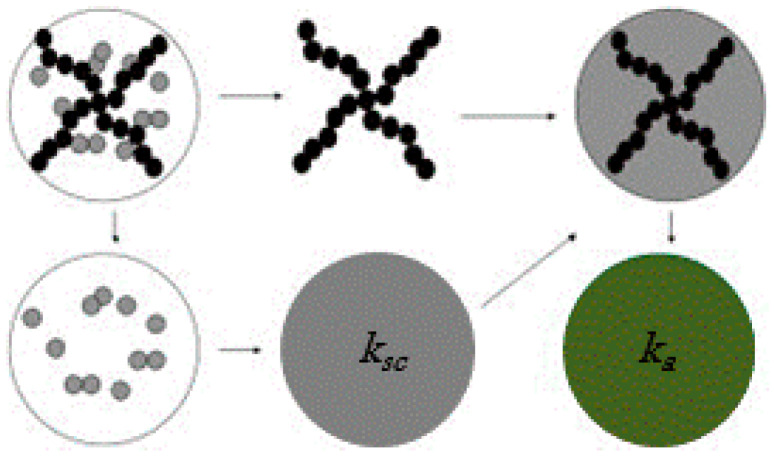
Schematic picture of the aggregate structure formed by nanoparticles arranged in a linear chain (**black**) and sidechains (**gray**). The conductivity of the whole aggregate (*k_g_*) is calculated by considering the conductivity of the backbone nanoparticles embedded in a medium with an effective conductivity *k_sc_*, due to the surrounding presence in the fluid of the sidechain nanoparticles. Adapted with permission from [231], Copyright 2006, AIP Publishing.

In this section, we report recent and significant results on NFs based on titania nanoparticles dispersed in water, focusing our interest on the effect of the nanoparticle aggregate structure on the thermal conductivity enhancement. Indeed, in such systems, much experimental evidence shows that a diffusion-limited colloid aggregation process [236,237] occurs, leading to the formation of fractal nanoparticle aggregates, which can be described as being composed of the primary backbone structure of linear chains plus a secondary structure of side chains (dead ends) [236,237]. To model the thermal conductivity of such fractal aggregates, Prasher et al. [231] proposed a three-level homogenization model, schematized in Figure 11, where the conductivity is calculated by considering the backbone immersed in a medium with an effective conductivity given by the contribution of the dead ends immersed in the liquid matrix.

In [231], the contribution of dead ends *k_sc_* is calculated using the Bruggeman model based on the EMA [202], which gives

(8) $$\frac{\left(1-\varphi_{SC}\right)\left(k_{l}-k_{sc}\right)}{\left(k_{l}+2k_{sc}\right)}+\frac{\varphi_{SC}\left(k_{p}-k_{sc}\right)}{\left(k_{p}+2k_{sc}\right)}=0$$

where *k_l_* and *k_p_* are the thermal conductivities of the fluid and particle, respectively, and $\varphi_{sc}$ is the volume fraction of particles belonging to dead ends, related to the volume fraction of particles in the aggregate $\varphi_{in}$ and in the backbone linear chains and $\varphi_{lc}$ by

(9) $$\varphi_{sc}=\varphi_{in}-\varphi_{lc}$$

According to the general description of fractal aggregates, these quantities can be calculated from

(10) $$\varphi_{in}={\left(\frac{R_{a}}{r_{p}}\right)}^{d_{f}-3}$$

and

(11) $$\varphi_{lc}={\left(\frac{R_{a}}{r_{p}}\right)}^{d_{l}-3}$$

where *d_f_* and *d_l_* are the so-called fractal dimension and chemical dimension of the aggregate, respectively, *R_a_* is the gyration radius (i.e., the radius of the embedding sphere), and *r_p_* is the radius of the nanoparticles (here assumed spherical). The fractal dimension and the chemical dimension specify the geometry of the aggregate: *d_f_* changes from 1 when all the nanoparticles are organized in a linear structure to 3 for spherical aggregates. In the case of TiO_2_ nanoparticles dispersed in water, *d_f_* has been measured to be in the range from 1.7 to 2.5 [238]. Analogously, the chemical dimension *d_l_* ranges from 1, when all the nanoparticles are structured in side chains, to *d_f_*, in which case all the nanoparticles are arranged in the backbone. 

The thermal conductivity of the aggregate as a whole can now be calculated using a model developed for randomly oriented cylindrical particles [204] representing the backbone linear chains immersed in the effective medium due to the side chains, giving

(12) $$k_{a}=k_{sc}\frac{3+\varphi_{lc}\left[2\beta_{11}\left(1-L_{11}\right)+\beta_{33}\left(1-L_{33}\right)\right]}{3-\varphi_{lc}\left[2\beta_{11}L_{11}+\beta_{33}L_{33}\right]}\;\;\;\;\;$$

where *L_ii_* and *β*_ii_ are geometrical factors strictly depending on the aspect ratio and on the thermal conductivities of the side chain nanoparticles and the base fluid [204]. Finally, the NF thermal conductivity *k* is calculated with the Maxwell-Garnett model [200] using the whole aggregate conductivity (12) and the volume fraction $\varphi_{a\;}$:

(13) $$\frac{k}{k_{l}}=\frac{\left(k_{a}+2k_{l}\right)+2\varphi_{a}\left(k_{a}-k_{l}\right)}{\left(k_{a}+2k_{l}\right)-\varphi_{a}\left(k_{a}-k_{l}\right)}$$

**Figure 12:**
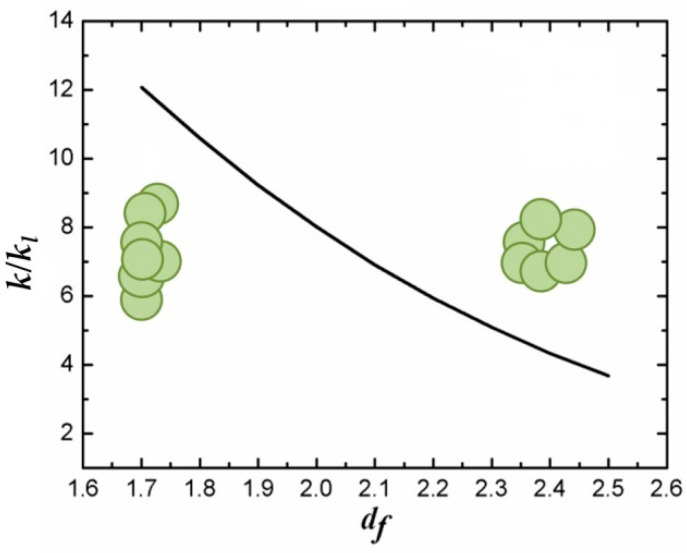
Influence of the fractal dimension on the thermal conductivity enhancement in TiO_2_-based NFs using Equation (13) and the parameters described in [220]. Adapted with permission from [220], Copyright 2014, Wiley and Sons.

The introduction in the model of terms relative to different structural nanoparticle arrangements allows us to better predict the experimental thermal conductivity enhancements than other models accounting for the aggregation effects [232,239]. Indeed, the data reported in Figure 12, obtained by expression (13), based exclusively on the conduction mechanism, show that spherical aggregates appear to be detrimental to the thermal conductivity enhancement, while linear chained structures, providing preferential conduction paths (percolation effect), promote the thermal conductivity enhancements with values over 10% [230,240,241].

The overall thermal conductivity of NFs based on fractal aggregates expressed by relation (13) was also validated by Prasher et al. [231] using Monte Carlo (MC) simulations. The large predicted difference of about 12% between the thermal conductivity enhancements of fractal aggregates compared to well-dispersed nanoparticles confirmed the strong effect of the structure (in terms of fractal and chemical dimensions) and size of the aggregates on the thermal conduction.

The effective role of the aggregate structure on heat conduction was experimentally demonstrated by Philip et al., who used a colloidal suspension of magnetically polarizable Fe_2_O_3_ nanoparticles [242]. This work showed that, when an external magnetic field induced linear chain aggregation of the nanoparticles in the fluid, an extraordinary thermal conductivity enhancement (300%) was observed at a low nanoparticle volume fraction (0.2%). In titania NFs, with the same nanoparticle volume fraction, the presence of linear aggregates was demonstrated to increase the thermal conductivity up to 5%. The model proposed by Prasher et al. [231] was successfully used to interpret the thermal conductivity enhancements from 2% to 6%, measured by varying the nanoparticle volume fraction from 0.1% to 0.6% [220], using a fractal dimension equal to 2.1, which is in good agreement with other results obtained for same aggregates [243,244].

Many studies have shown that the aggregate structure is influenced by different factors (drivers), generally related to the NF’s preparation parameters and to the temporal evolution of the aggregation state occurrences. For example, an optimum sonication time was found to induce the formation of small aggregates with linear structures and hence obtain a maximum thermal conductivity enhancement [245,246,247,248]. On the other hand, another way to induce the formation of linear chains of nanoparticles is to vary the pH value [190,248,249]. Although most of the experiments on thermal conduction reported in the literature were conducted at pH values ranging from 7 to 9 [246], a huge thermal conductivity enhancement of 20% was observed near the isoelectric point (pH = 4.5 in water) [248] in NFs containing 3% of TiO_2_ volume fraction. At this pH value, the repulsive forces of the nanoparticles are absent, and the particles come closer due to van der Waals interactions, forming irregular aggregates with linear structures, thus improving the heat conduction. On the other hand, the aggregate mass increase can cause a drastic reduction of the measured thermal conductivity for the precipitation of the aggregates. To overcome the problem of the aggregates’ sedimentation and to improve the stability of the NF, it is possible to modify the surfaces of the nanoparticles with different surfactants. In this way, greater stability of the NF and similar thermal conductivity enhancements at low nanoparticle volume fractions (<0.5%) were obtained when compared to unmodified nanoparticles [250,251,252], while at higher particle concentrations, the presence of surfactants leads to a significant enhancement in thermal conductivity, minimizing the formation of large spherical aggregates [251,253].

All the experimental results reported above were obtained at room temperature and generally deviated from the theoretical predictions here described within errors ranging from ±3% to ±5% [240]; however, when the experimental temperature was raised to 70 °C, larger deviations were observed [254,255]. To account for the temperature effects, Kundan et al. [240] introduced a term describing the micro-convection contribution induced by the nanoclusters’ Brownian motion (BM) in Equation (13), obtaining

(14) $$\frac{k}{k_{l}}=\frac{\left(k_{a}+2k_{l}\right)+2\varphi_{a}\left(k_{a}-k_{l}\right)}{\left(k_{a}+2k_{l}\right)-\varphi_{a}\left(k_{a}-k_{l}\right)}\left(1+AR_{eB}^{m}P_{r}^{0.333}\varphi_{a}\right)$$

where *A* and *m* are parameters whose values depend on the nature of the NF (for metal oxide *A* ≈ 5 × 10^4^ and *m* ≈ 2.5%) [231,256], *P_r_* is Prandtl’s number [254], and *R_eB_* is the Brownian Reynold number [257,258] accounting for the base fluid state, nanoparticles and fluid density and size. Equation (14) predicts that the contribution of the BM of the aggregates to the thermal conductivity enhancement at room temperature is limited, while it becomes significant when increasing the temperature up to 70 °C because of the nanocluster’s thermal excitations and, in some cases, for the breaking of large clusters into smaller ones.

Despite the different measurement conditions used, many experimental investigations on titania NFs showed an evident increase in the thermal conductivity enhancement when varying the temperature from 20 °C to 70 °C [190,240,250,259,260,261], in accordance with these predictions. Nevertheless, there is no unanimous agreement on the temperature dependence of the thermal conductivity, as demonstrated by Duangthongsuk et al. [262], who observed a decrease with the temperature rise, and by Turgut et al. [219], who showed a constant behavior.

Although in most of the published studies on NFs, the size of TiO_2_ nanoparticles ranges from 20 to 40 nm, several works have indicated that at the same NF status and composition (base fluid, pH, temperature, nanoparticle volume fraction), the nanoparticle size impacts the NF thermal conductivity. In fact, very small particles (<10 nm) tend to aggregate, forming large compact clusters [263,264], which as predicted by Equation (13) leads to a reduction of the thermal conductivity enhancement, limiting the contribution of the BM to the heat transport [231]. On the other hand, in the presence of these small particles, at a volume fraction higher than 1%, the nanoaggregate–fluid interface plays a significant role due to the possible formation of a well-defined ordered layer with a solid-like structure (nanolayer) [235,265,266,267,268]. For this reason, Feng et al. [235] proposed a model combining the effects of the nanolayer’s and nanoaggregate’s presence on the heat conduction, considering the role of nanoparticle size and volume fraction, the thickness of the interfacial nanolayer, the thermal conductivities of the cluster and the base fluid. The model predictions are reasonable and in good agreement with the available experimental data on different metal oxide-based NFs.

The significant role of linearly structured aggregates on the thermal conduction mechanisms is also reflected in the large thermal conductivity enhancements observed in NFs containing non-spherical shape titania nanoparticles. Indeed, according to the HC model expressed by Equation (7), a higher thermal conductivity enhancement (about 3%) is predicted for rod-shaped titania nanoparticles compared to spherical ones [201]. However, some experimental evidence [269,270] reveals an even higher effect of nanoparticle shapes on thermal conduction than predicted. In particular, Murshed et al. [269] reported thermal conductivity enhancements of 20% for 1% rod-shaped (10 nm × 40 nm) nanoparticles volume fractions compared to 10% obtained for spherical ones (15 nm). To better interpret these high thermal conductivity enhancements obtained for particles with high aspect ratios, Wei et al. [271] proposed a new analytical expression based on the HC model, considering the effect of the aggregation and hence substituting the nanoparticle volume fraction and thermal conductivity with terms relative to the presence of fractal aggregates as described above. The comparison of their theoretical results with those obtained experimentally [269] showed a very good agreement, confirming the model’s validity.

Accordingly, nanotubes should also provide an excellent model material for examining the effect of the particle shape on the thermal conduction of NFs [272,273,274]. However, despite the large thermal conductivity enhancements measured in carbon nanotube-based NFs [275,276], TiO_2_ nanotubes dispersed in water showed lower values. For instance, Chen et al. [277] synthesized strongly protonated TiO_2_ (titanate) nanotubes using the hydrothermal method [278] and observed a small thermal conductivity enhancement of about 3% with 0.6% nanotubes (10 nm × 100 nm) loading. Despite this small thermal conductivity enhancement, the measured values are higher than those predicted for well-dispersed non-spherical nanoparticles by the HC model and that observed in NF based on spherical nanoparticles. However, these results confirm the significant role of the nanoparticle shape on the thermal conduction enhancement; in particular, its crucial effect on the convective heat transfer and the clustering mechanism [277].

The results reported in this section highlight the significant role of the structure of fractal aggregates and the morphology of titania nanoparticles on the thermal conduction in TiO_2_-based NFs. Indeed, several experimental results showed that aggregates with a linear structure or nanoparticles with high aspect ratios could enhance thermal conductivity. In addition, among the numerous theoretical approaches present in the literature to interpret the high thermal conductivity enhancements observed experimentally, those based on the percolation effect due to the presence of fractal aggregates lead to the justification of the observed behavior of metal oxide-based NFs, especially with respect to the temperature and the nanoparticles’ size and volume fraction dependence.

On the other hand, preparing NFs with a controlled size and structure of the aggregates is a crucial task and, due to difficulty in obtaining reproducible measurements, the experimental results are often controversial, making the interpretation of thermal mechanisms difficult.

Since the NFs are considered a valid alternative to simple fluids in many heat transfer applications, further and more accurate studies are required to better understand the role of the different NF parameters on heat transfer and to identify the mechanisms responsible for the thermal conductivity enhancement.

## 5. Conclusions

In this work, we reviewed three important applications of nanophase TiO_2_ in the fields of energy production and management, with the scope of evidencing the importance of the material morphology and structure in optimizing the functional properties for each use. This approach allowed us to discuss in the same light photovoltaic energy conversion, hydrogen production by water splitting and thermal energy management by nanofluids. We reviewed the functionalities necessary for the use of the nanomaterial in each case, starting from the widely studied basic morphology, consisting of aggregated nanobeads, and arriving at 1D or hierarchical structures. Where possible, we described the models relating the nanomaterial properties to the process efficiency; notably, it appears that nanofluid modeling is the more developed field. Reasons for this might be related to the abundance of related studies on colloids and suspensions and also to the simpler functionality, i.e., thermal energy transport, required for the application. On the other hand, photovoltaics and water splitting applications could benefit from a unified modeling approach, which is however still lacking in the literature.

## Data Availability

This study did not report any original data.
